# The Use of Vancomycin Versus Teicoplanin in Treating Febrile Neutropenia: A Meta-Analysis and Systematic Review

**DOI:** 10.7759/cureus.15269

**Published:** 2021-05-27

**Authors:** Jasmeet Kaur, Tanveer Mir, Priyadarshini Dixit, Mohammad Uddin, Saritha Kadari, Yi Lee, Prateek Lohia, Rafiullah Khan

**Affiliations:** 1 Internal Medicine, Saint Joseph Mercy Oakland Hospital, Pontiac, USA; 2 Internal Medicine, Wayne State University Detroit Medical Center, Detroit, USA; 3 Urology, Chung Shan Medical University, Taichung, TWN; 4 Division of Hematology-Oncology, Department of Internal Medicine, University of Cincinnati Medical Center, Cincinnati, USA

**Keywords:** vancomycin, teicoplanin, febrile neutropenia, outcomes

## Abstract

Background and objective

The efficacy of vancomycin vs. teicoplanin for the successful treatment of febrile neutropenia (FN) has been a subject of debate in the medical community. In light of this, we performed a systematic review and meta-analysis to compare these two medications in the treatment of patients with FN in terms of treatment success and adverse events.

Data source and study design

We conducted a search of major electronic databases [MEDLINE (PubMed, Ovid), Google Scholar, clinicaltrial.org], which returned 10 studies with 1,630 patients (vancomycin: 788; teicoplanin: 842) for analysis. An unadjusted odds ratio (OR) with a 95% confidence interval (CI) was calculated for all studies, as well as separate sub-analyses of randomized controlled trials (RCTs) and retrospective studies.

Results

The average age of patients ranged from 37 to 57 years in the vancomycin group and 31 to 57 years in the teicoplanin group (n=9 studies). Over half of the patients in both groups were male (vancomycin: 55.6%; teicoplanin: 57.7%; n=9 studies). Both overall evaluation and sub-analyses revealed that both treatments were comparable in terms of treatment success, nephrotoxicity, and red man syndrome. The vancomycin group was more likely to develop skin rashes (OR: 2.49; 95% CI: 1.28-4.83). The heterogeneity for all analyses ranged from 0-47.4%.

Conclusion

Our analysis showed that vancomycin and teicoplanin showed comparable results in terms of successful treatment of FN. Adverse effects such as nephrotoxicity and red man syndrome were also comparable between the two treatment groups.

## Introduction

The Infectious Diseases Society of America (IDSA) defines febrile neutropenia (FN) as a single oral temperature of ≥38.3 °C (101 °F) or a temperature of ≥38.0 °C (100.4 °F) sustained over a period of one hour [1]. Neutropenia is defined as an absolute neutrophil count (ANC) of <1,500 or 1,000 cells/microL, severe neutropenia as an ANC of <500 cells/microL, and profound neutropenia as an ANC of <100 cells/microL [1]. The risk for infections is higher in patients with ANC of <500 cells/microL and prolonged neutropenia (>7 days) [1]. IDSA has classified FN into microbiologically documented infection based on a microbial focus of infection and an associated pathogen. Clinically documented infection is FN with a clinical focus but without isolating an associated pathogen, and unexplained fever is FN without a microbial or clinical focus [1].

The most common factors contributing to the development of neutropenic fever in cancer patients include the direct effects of chemotherapy on mucosal barriers and breeches in the host defenses, which increase the risk of invasive infection. The common pathogens include *Pseudomonas aeruginosa*, *Staphylococcus (Staph) epidermidis*, *Staph aureus*, *Streptococcus viridians*, and *enterococci* [2].

Timely identification of the development of neutropenic fever is crucial to initiate prompt empiric therapy to avoid progression to sepsis that could lead to fatal outcomes. Systemic treatment with empiric antimicrobial agents with broad-spectrum antibiotic coverage includes antipseudomonal agents, such as cefepime, carbapenem, and piperacillin/tazobactam, initiated after blood cultures are drawn. The administration of empiric antibacterial therapy for sepsis should occur within 60 minutes of presentation as per the Surviving Sepsis 3 guidelines [2,3]. The type of antibiotic choice also depends on the patient’s immunocompromised status, prior history of infections due to antibiotic-resistant organisms, and initial presentation. The standard empiric antibiotic therapy for FN usually involves monotherapy with *Pseudomonas* coverage. However, if the initial presentation is complicated by hemodynamic instability, pneumonia, or cellulitis, the addition of antibiotics with Gram-positive cocci may be considered, which includes the administration of an antibiotic that covers Methicillin-resistant *Staphylococcus aureus* (MRSA) (vancomycin, teicoplanin) and Gram-negative bacteria (aminoglycoside or fluoroquinolone) [1]. Teicoplanin is also effective for treating vancomycin-resistant *enterococci*. While teicoplanin is not approved in the United States, it is available in Asian and European countries [4].

Vancomycin and teicoplanin have been used in the treatment of neutropenic fever, and their various adverse effects and efficacy profiles have been described in the literature. In this meta-analysis and systematic review, we engage in a comparative analysis to evaluate the efficacy and adverse effect profiles of vancomycin versus teicoplanin in patients with FN.

## Materials and methods

Search strategy

**Figure 1:**
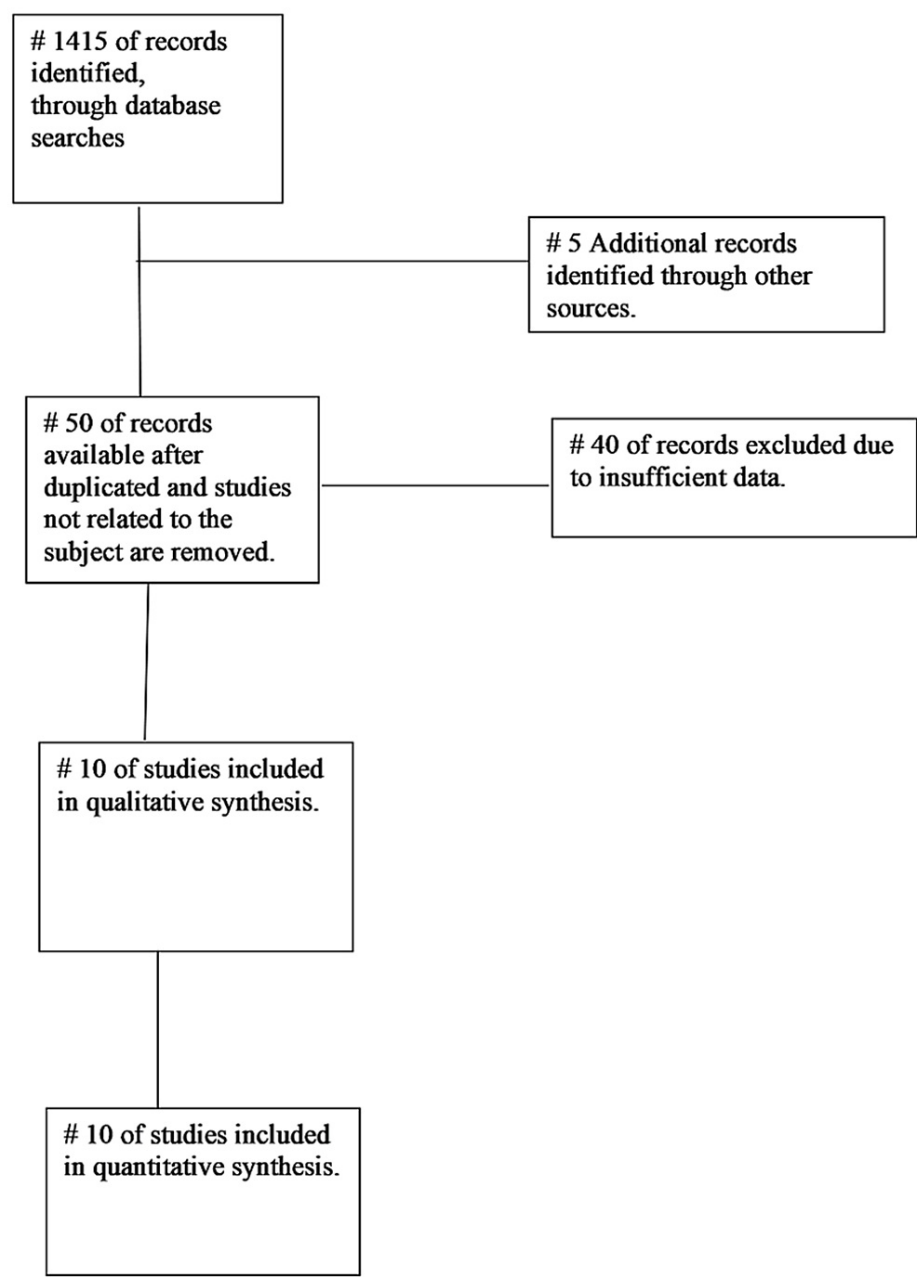
PRISMA flow chart depicting the selection of studies PRISMA: Preferred Reporting Items for Systematic Reviews and Meta-Analyses

Electronic databases including MEDLINE (PubMed, Ovid), Google Scholar, and clinicaltrial.org were searched using a combination of Medical Subject Headings (MeSH) terms and key terms like "vancomycin" AND " Teicoplanin" AND "Febrile neutropenia" AND "treatment" AND” Outcomes”. A cross-reference check was performed on this topic for previously published articles. The eligibility of the studies was checked by two independent authors (TM, JK). Articles were initially screened at the level of titles and abstracts. The full texts of potentially relevant articles were perused by the two independent authors (TM, JK). Disagreements were resolved through consensus [5]. The Preferred Reporting Items for Systematic Reviews and Meta-Analyses (PRISMA) guidelines were followed to obtain studies for quantitative analysis (Figure 1).

Inclusion and exclusion criteria

The inclusion criteria of all studies were uniform in nature. Studies that involved patients who were >14 years of age, comparing vancomycin with teicoplanin in treating FN (defined as a body temperature of >38.0 °C persisting for an hour or a single measured temperature of ≥38.5 °C combined with neutropenia) in terms of treatment success and adverse events were included. Treatment success was defined as the resolution of fever and clinical signs of infection and the eradication of the infecting microorganisms without any changes in allocated antibiotics. Adverse effects from the medications included red man syndrome, skin rashes, and nephrotoxicity. The outcomes in patients during the period of their hospitalization were evaluated, and hence prolonged follow-up was not required. Exclusion criteria included single-arm studies, studies with insufficient/duplicate data, as well as case reports, conference papers, and review articles.

Objective

The objective of this study was to evaluate the efficacy and toxicity profiles (nephrotoxicity, skin toxicity, and red man syndrome) between vancomycin and teicoplanin in patients with FN with a history of malignancy (acute myeloid leukemia, non-Hodgkin’s lymphoma, multiple myeloma, and a history of bone marrow transplant). For this purpose, we searched for prospective and retrospective studies comparing the efficacy and toxicity profiles of vancomycin vs. teicoplanin in patients with FN.

Outcome measures

The primary outcome measure was to compare the efficacy between vancomycin and teicoplanin in patients with FN. And the secondary outcome measure was to compare toxicities such as nephrotoxicity, red man syndrome, and skin toxicity in patients with FN treated by vancomycin vs. teicoplanin.

Search and data extraction

The data of the included studies were collected on a spreadsheet and verified by a third author. Baseline characteristics, clinical presentations, types of malignancy, bone marrow transplant status, empiric antibiotics, toxicity, follow-up data, and clinical outcomes of all patients were recorded. The primary outcome measure was treatment success, and secondary outcome measures were nephrotoxicity, skin rashes, and red man syndrome.

Statistical analysis

Statistical analyses were performed using the random effect restricted maximum-likelihood (REML) model to calculate an unadjusted odds ratio (OR). "The random-effects model assumes that the studies included in the meta-analysis are a random sample of hypothetical study populations" [6]. The estimated effect size was reported as a point estimate and 95% confidence interval (CI) for all studies and separate sub-analyses of randomized controlled trials (RCTs) as well as retrospective cohort studies. A p-value of <0.05 was considered statistically significant. The Higgins I-squared (I^2^) parameter was used to evaluate the heterogeneity of included studies [7]. I^2^ values of 50% or less corresponded to low to moderate variation, and those of 75% or higher indicated a considerable amount of heterogeneity. Publication bias was assessed using a graphical presentation, using Harbord’s weighted linear regression. All included articles were screened for five different types of bias (selection, performance, detection, attrition, and reporting bias) to assess methodological quality and were evaluated as per the Newcastle-Ottawa Scale for retrospective studies. The STATA software version 16.1 (StataCorp, College Station, TX) was used to perform all statistical analyses.

## Results

Search results and study characteristics

The initial search identified 1,420 articles; after excluding duplicates and irrelevant articles, 50 studies were deemed relevant for full-text review (Figure 1). Of those, 40 articles were excluded due to insufficient data. Finally, 10 studies were found suitable for quantitative analysis [8-17]: four retrospective cohort studies [11,14,15,17] and six RCTs [8-10,12-13,16] (Table 1).

**Table 1:** Characteristics of studies comparing outcomes of vancomycin versus teicoplanin ^Ϯ^Reported number of episodes RCT: randomized controlled trial; RCS: retrospective cohort study; V: vancomycin; T: teicoplanin; NA: not available

Author and year	Study type	Blinding	Study number	Number of patients per arm		Average age of patients in years, mean or median (range)		Male gender, n		Empiric antibiotics	Baseline creatinine, mg/dl		Duration of treatment, days		Nephrotoxicity		Red man syndrome		Skin rash		Successful response	
				V	T	V	T	V	T		V	T	V	T	V	T	V	T	V	T	V	T
Cony-Makhoul et al., 1990 [16]	RCT	Not defined	59^Ϯ^	35	24	45 (16-80)	51.5 (17-69)	17	15	Ceftazidime	NA	NA	NA	NA	0	0	0	0	0	0	21	13
Chow et al., 1993 [8]	RCT	Double-blinded	50	25	25	38 (20-76)	40 (19-68)	15	11	Piperacillin, tobramycin	0.76 (.51-1.29)	0.8 (0.52-1.26)	NA	NA	10	2	1	0	1	0	21	23
Menichetti et al., 1994 [9]	RCT, multi-center	Open-label	527	252	275	42 (14-72)	44 (14-78)	132	158	Ceftazidime, amikacin	NA	NA	12 (1-59)	12 (3-40)	2	4	NA	NA	15	4	190	216
Nucci et al., 1998 [10]	RCT	Not defined	90	44	46	37 (12-72)	31 (13-71)	37	28	Ceftazidime, amikacin	NA	NA	10	10	2	2	0	1	3	2	23	25
Vázquez et al., 1999 [12]	RCT	Not defined	76	38	38	47	51	21	15	Piperacillin, tazobactam + amikacin	NA	NA	NA	NA	1	1	4	0	NA	NA	17	18
Bucaneve et al., 1999 [11]	RCS		527	252	275	NA	NA	NA	NA	Ceftazidime, amikacin	NA	NA	12	11	2	4	NA	NA	15	4	190	216
D'Antonio, et al., 2003 [13]	RCT	Double-blinded	124	61	63	37.2 (21.6-52.8)	41.5 (24.9-58.1)	29	29	Ceftazidime, amikacin	NA	NA	11.4 ± 2.8	12.2 ± 4.2	2	1	NA	NA	5	5	56	55
Hahn-Ast et al., 2008 [14]	RCS		91	49	42	57	57	26	32	Piperacillin, tazobactam	0.9 (0.8-1)	0.9 (0.8-1)	8	10	15	7	NA	NA	NA	NA	29	32
Kato-Hayashi et al., 2019 [17]	RCS		29	13	16	53 (32-63)	48 (36-64)	7	8	Cefepime/carbapenem or piperacillin, tazobactam	0.49 ± 0.17	0.52 ± 0.14	11	14	6	0	NA	NA	NA	NA	6	13
Ohata et al., 2020 [15]	RCS		57	19	38	53 (23-72)	49 (17-66)	7	25	Cefepime/carbapenem or piperacillin, tazobactam	0.4 (0.3-0.6)	0.5 (0.4-0.6)	13	15.5	NA	NA	NA	NA	NA	NA	19	35
Total	-	-	1,630	788	842	-	-	291	321	-	-	-	-	-	40	27	5	1	39	15	572	646

The included studies involved a total of 1,630 patients (vancomycin: 788; teicoplanin: 842). The average age reported ranged from 37 to 57 years in the vancomycin group and 31 to 57 years in the teicoplanin group (n=9 studies). Nine studies reported the gender of the subjects; over half of the patients in both groups were male [vancomycin: 55.6% (291/523); teicoplanin: 57.7% (321/556)]. Acute leukemia was the condition with the highest rate of prevalence in both groups, and its incidence was comparable between the two groups (86.12% vs. 84.13%) (Table 2).

**Table 2:** Characteristics of included trials V: vancomycin; T: teicoplanin; NA: not available

Author and year	Acute leukemia		Chronic leukemia		Non-Hodgkin’s lymphoma		Myeloma		Bone marrow transplant	
	V	T	V	T	V	T	V	T	V	T
Cony-Makhoul et al., 1990 [16]	33	20	NA	NA	NA	NA	1	2	NA	NA
Chow et al., 1993 [8]	14	14	8	10	NA	NA	NA	NA	12	16
Menichetti et al., 1994 [9]	215	235	NA	NA	NA	NA	NA	NA	22	29
Nucci et al., 1998 [10]	33	32	NA	NA	17	16	NA	NA	3	8
Vázquez et al., 1999 [12]	10	10	NA	NA	7	10	3	3	18	15
Bucaneve et al., 1999 [11]	252	275	NA	NA	NA	NA	NA	NA	NA	NA
D'Antonio, et al., 2003 [13]	48	39	2	4	9	15	2	4	NA	NA
Hahn-Ast et al., 2008 [14]	42	38	NA	NA	NA	NA	NA	NA	NA	NA
Kato-Hayashi et al., 2019 [17]	NA	NA	NA	NA	NA	NA	NA	NA	13	16
Ohata et al., 2020 [15]	NA	NA	NA	NA	NA	NA	NA	NA	19	38
Total	647	663	10	14	33	41	6	9	87	122

Primary outcome

**Figure 2:**
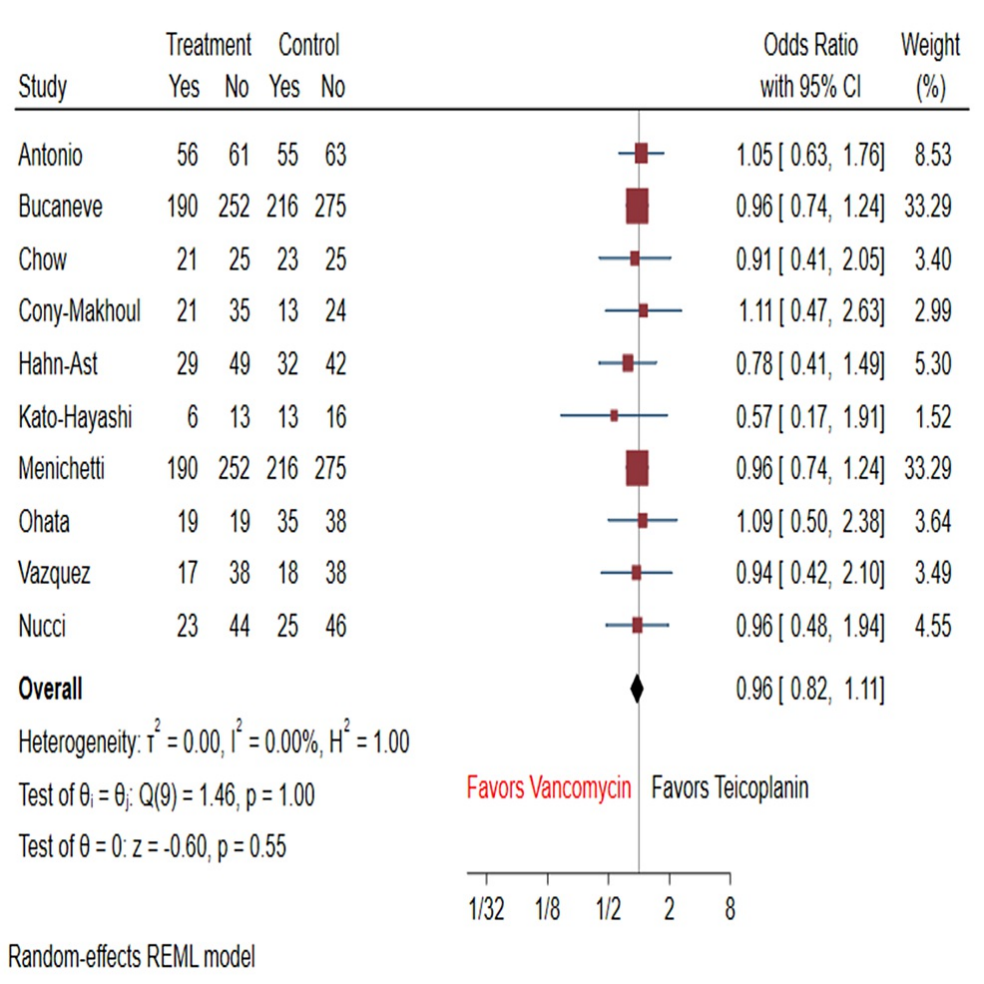
Forest plot comparing odds ratio of success rates for vancomycin vs. teicoplanin Results were comparable (subgroup analysis for RCTs did not show any differences) [8-17] Treatment group: vancomycin group; control group: teicoplanin group RCT: randomized controlled trial

Treatment success was achieved in 72.4% (n=572) of patients on vancomycin and 76.5% (n=646) of patients on teicoplanin. These results were comparable between the two groups when all studies were taken into account (OR: 0.96; 95% CI: 0.82-1.11; I^2^=0%), as well as when assessing RCTs (OR: 0.98; 95% CI: 0.80-1.20; I^2^=0%) and retrospective cohort studies (OR: 0.93; 95% CI: 0.74-1.16; I^2^=0%) separately in the form of sub-analyses (Figure 2).

Secondary outcomes

**Figure 3:**
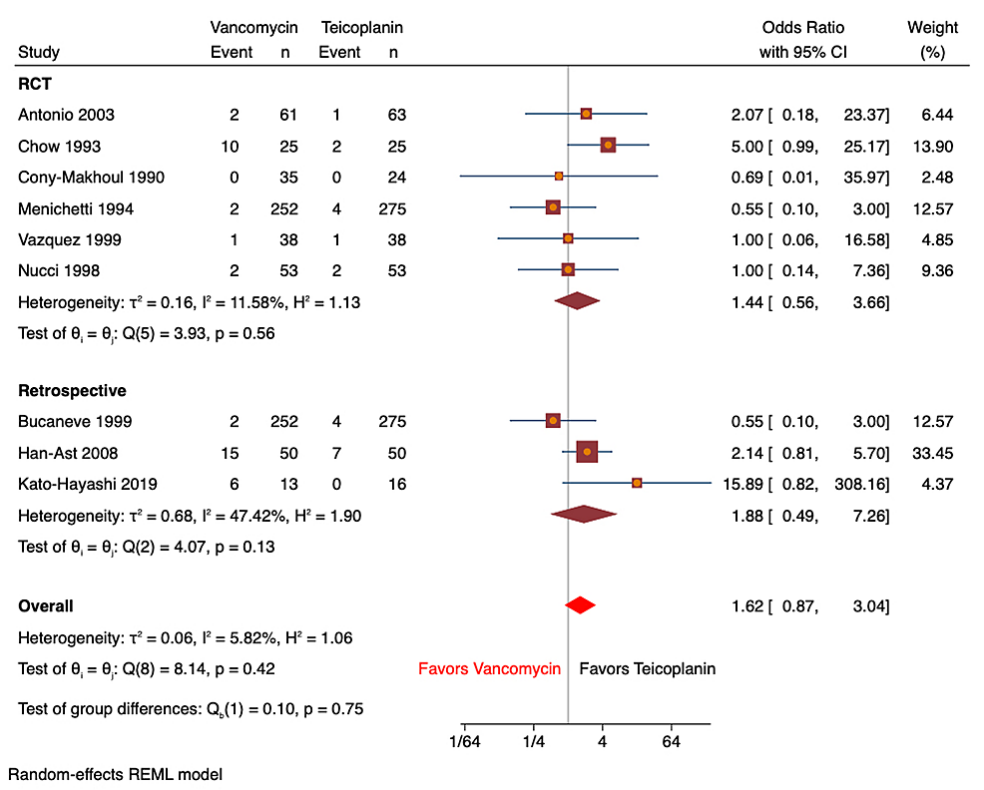
Forest plot comparing odds ratio of nephrotoxicity for vancomycin vs. teicoplanin Results were comparable (subgroup analysis for RCT did not show any differences) [8-14,16,17] RCT: randomized controlled trial

**Figure 4:**
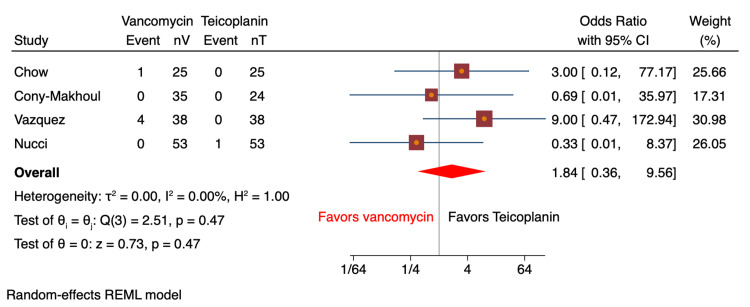
Forest plot comparing odds ratio of red man syndrome for vancomycin vs. teicoplanin Results were comparable between the two groups [8,10,12,16]

**Figure 5:**
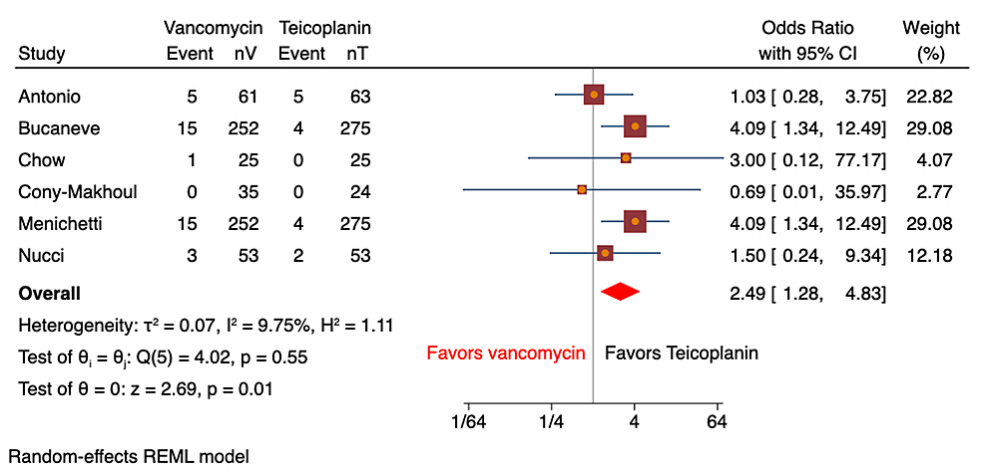
Forest plot comparing odds ratio of skin rash rates for vancomycin vs. teicoplanin Higher rates of skin rashes were observed in the vancomycin group [8-11,13,16]

Nephrotoxicity as an adverse effect was reported in nine studies (n=1,598 patients; vancomycin: n=779, teicoplanin: n=819) based on the elevation in baseline creatinine [8-14,16,17]. Nephrotoxicity was reported in 40 (5.1%) patients on vancomycin vs. 21 (2.6%) patients on teicoplanin. There were no significant differences noted between the groups pertaining to these findings (OR: 1.62; 95% CI: 0.87-3.04; I^2^=5.8%; Figure 3). Sub-analyses for RCT (OR: 1.44; 95% CI: 0.56-3.66; I^2^=11.6%) and retrospective cohort studies (OR: 1.88; 95% CI: 0.49-7.26; I^2^=47.4%) showed similar results. Heterogeneity was 0% for all analyses. Red man syndrome was analyzed in a total of four studies (n=291 patients; vancomycin: n=151, teicoplanin: n=140) [8,10,12,16]; 3.3% (n=5) of patients in the vancomycin group had red man syndrome, and the teicoplanin had 0.7% (n=1). The results were not statistically significant (OR: 1.84; 95% CI: 0.36-9.56; I^2^=0%; Figure 4). Skin rash was reported in six studies (n=1,393; vancomycin: n=678, teicoplanin: n=715) [8-11,13,16]. Skin rashes were observed in 5.8% (n=39) of patients on vancomycin compared to 2.2% (n=15) of patients on teicoplanin. The pooled analysis revealed a significant association, with vancomycin patients found more than twice likely to develop a skin rash compared to patients on teicoplanin (OR: 2.49; 95% CI: 1.28-4.83; I^2^=9.8%; Figure 5).

Publication bias

**Figure 6:**
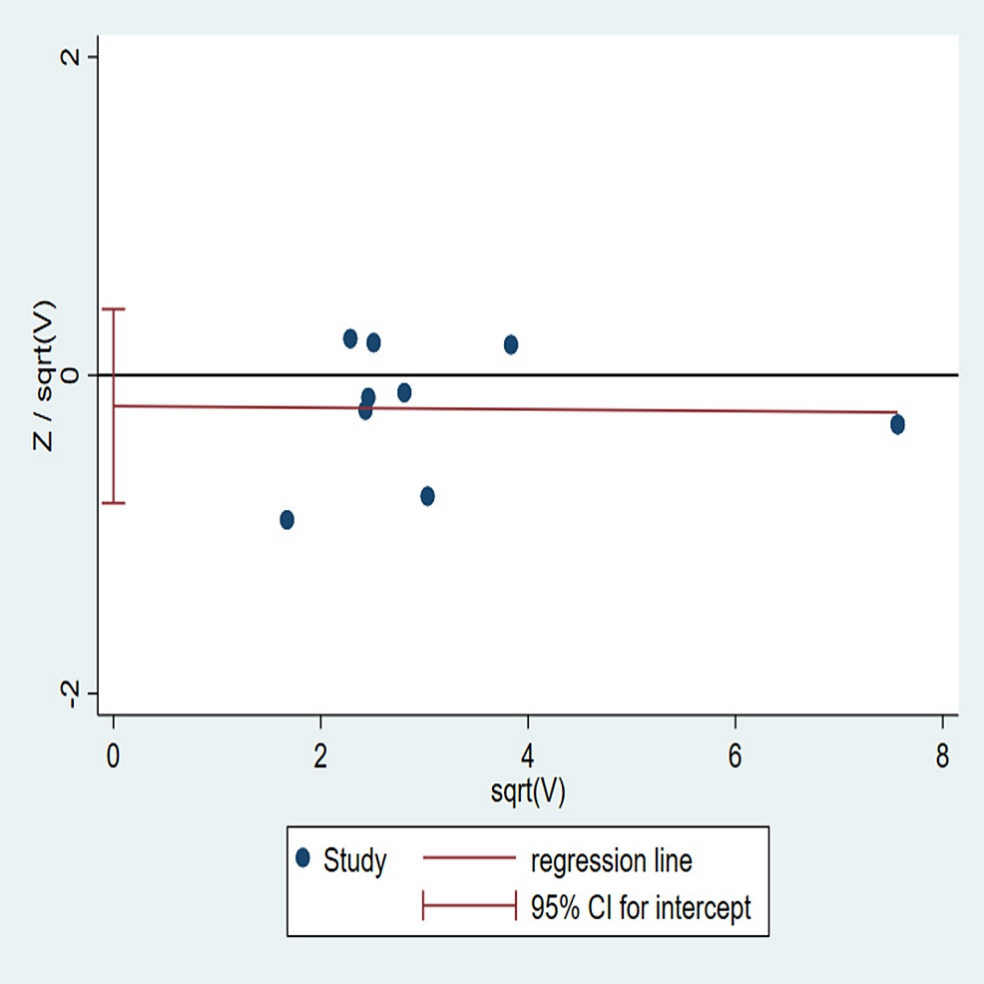
Harbord’s weighted linear regression The data was run on 10 studies; some studies were overlapping [8-17]. Funnel is widely used for assessing the publication bias between the studies

**Figure 7:**
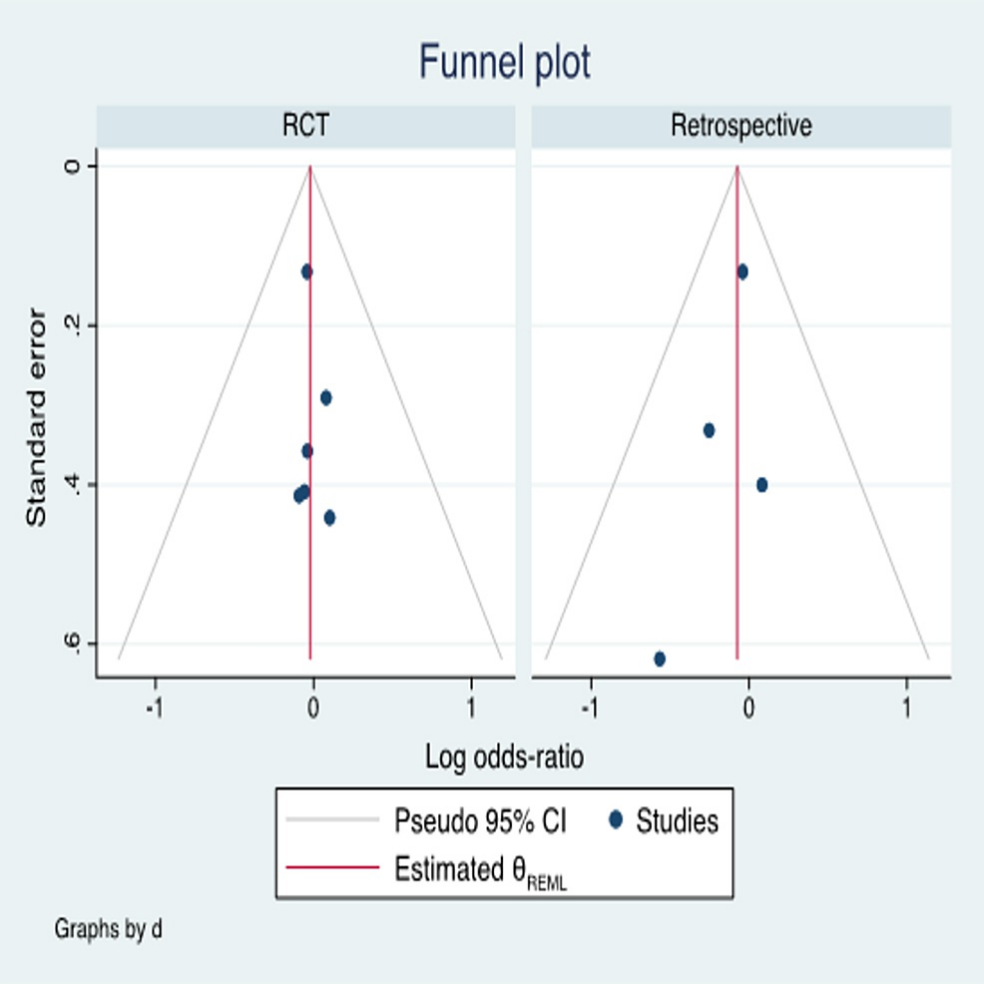
Graphic evaluation for bias among the subgroups No evident bias was seen RCT: randomized controlled trial

The publication bias was illustrated graphically with Harbord’s weighted linear regression [18]. "The vertical axis of the plot used standard error to estimate the sample size of the study, plotting large population studies on top and smaller at the bottom" [5]. "The horizontal spread reflected the power and effect size of the included studies" [5]. Harbord’s weighted linear regression indicated the absence of publication bias among the 10 studies [t=-0.08, p=0.48 (95% CI: -0.80-0.41)] (Figure 6). Publication bias was checked among the studies included in the subgroup analyses as well. No evident bias was noticed (Figure 7).

Quality of the included studies

There were four observational retrospective studies included in the analysis, and this posed some theoretical risk of selection bias due to a lack of randomization and allocation concealment. An adequate description of the study results helps in reducing concerns regarding reporting bias. The quality of randomized trials was calculated by the revised Cochrane risk-of-bias tool for randomized trials, and bias was found to be low (Table 3). The quality assessment was performed using the Newcastle-Ottawa Scale for observational studies. The quality of the studies was found to be moderate (≥7) (Table 4).

**Table 3:** Revised Cochrane risk-of-bias tool for randomized trials

Study	Risk of bias arising from the randomization process (selection bias)	Risk of bias due to deviations from the intended interventions (effect of assignment to intervention)	Risk of bias due to missing outcome data (attrition bias)	Risk of bias in the measurement of the outcome (detection bias)	Risk of bias in the selection of the reported results	Overall risk of bias judgment
D'Antonio, et al., 2003 [13]	Low	Low	Low	Low	Low	Low
Chow et al., 1993 [8]	Low	Low	Low	Low	Low	Low
Cony-Makhoul et al., 1990 [16]	Low	Low	Low	Low	Low	Low
Menichetti et al., 1994 [9]	Low	Low	Low	Low	Low	Low
Vázquez et al., 1999 [12]	Low	Low	SC	Low	Low	Low
Nucci et al., 1998 [10]	Low	Low	Low	Low	Low	Low

**Table 4:** Quality of observational studies assessed as per the Newcastle-Ottawa scale ☆: denotes one point for the given criteria

Study	Selection				Comparability	Exposure			Total score
	Adequate case definition	Representativeness of cases	Selection of controls	Definition of controls	Comparability of cohorts	Ascertainment of exposure	Same method of ascertainment	Non-response rate	
Bucaneve et al., 1999 [11]	☆	☆	☆	☆	-	☆	☆	☆	7
Hahn-Ast et al., 2008 [14]	☆	☆	☆	☆	☆	☆	☆	☆	8
Kato-Hayashi et al., 2019 [17]	☆	☆	☆	☆	-	☆	☆	☆	7
Ohata et al., 2020 [15]	☆	☆	☆	☆	-	☆	☆	☆	7

## Discussion

To the best of our knowledge, this meta-analysis and systematic review is the first of its kind to compare the effects of vancomycin with those of teicoplanin in FN patients. Our study found vancomycin and teicoplanin to be comparable in terms of therapeutic success and rates of adverse effects, except for skin rashes. However, rates of skin rashes were minimal in both groups. The results of this study align with those reported by other studies that compared the use of these medications in various study populations [4,19-22].

A multicenter prospective observational study conducted by Yoon et al. reported no differences in adverse events, efficacy, and overall mortality between patients on vancomycin and those on teicoplanin. The study focused on healthcare-associated methicillin-resistant *staph* infections [23]. A meta-analysis including both neutropenic and non-neutropenic patients by Svetitsky et al. did not reveal any differences in terms of efficacy and mortality outcomes between vancomycin and teicoplanin [4]. Also, another study by Cavalcanti et al. revealed no significant difference in efficacy for every suspected or proven infection [20].

In terms of adverse effects, red man syndrome, which is secondary to histamine release from antibiotic infusion, was comparable between the two treatment options in our study. Sahai et al. evaluated red man syndrome between vancomycin and teicoplanin, and the results showed higher rates associated with vancomycin compared to teicoplanin [24]. However, the cohort in this study was not restricted to neutropenic patients. Smith et al. reported higher rates of nephrotoxicity, red man syndrome, and rashes with vancomycin compared to teicoplanin in FN patients with a Hickman catheter, though the overall efficacy was comparable between the two treatment options [21]. Van der Auwera et al. evaluated vancomycin vs. teicoplanin in non-neutropenic immunocompromised patients [22]. The study revealed comparable efficacy between the two treatment options, while higher rates of adverse effects in terms of skin rash and nephrotoxicity were reported in the vancomycin group.

Our study showed no significant difference in the occurrence of nephrotoxicity between the two treatment options. Our findings are in line with those of Wood, who also did not reveal any differences in terms of nephrotoxicity between the two treatment options [19]. This could be attributed to the smaller number of patients with nephrotoxicity in the cohort (40 patients in the vancomycin group and 27 patients in the teicoplanin group), which precluded any statistically significant differences. Cavalcanti et al. revealed higher rates of nephrotoxicity in the vancomycin treatment group compared to teicoplanin in patients with proven and suspected infections [20]. Our study revealed higher rates of skin rashes with vancomycin compared to teicoplanin, which concurs with other studies in the literature [11,14,20].

Limitations

This study has some limitations, which are primarily a reflection of the limitations of the included studies. One such limitation was our inability to perform a stratified subgroup analysis based on the variable follow-up durations and different selection criteria. The inherent heterogeneity in the observational data could have led to some risks of bias; however, heterogeneity in all the analyses was found to be low to moderate. The inclusion of retrospective studies in our analysis also posed some risks of bias due to non-randomized assignments. However, the results for the subgroup analyses for RCTs and retrospective studies were found to be similar. This was essentially a study-level meta-analysis that had a limited ability to examine the source of heterogeneity, and we believe that a patient-level meta-analysis might provide additional evidence on the subject.

## Conclusions

Vancomycin and teicoplanin had comparable results in terms of treatment success in FN patients. The adverse effects including nephrotoxicity and red man syndrome were also comparable between the treatment groups. Since patients with toxicity constituted a very small segment of the study population, our study did not yield statistically significant results regarding this parameter, and further studies are required to gain more insight into this aspect. Vancomycin group had higher rates of skin rashes compared to patients on teicoplanin. Future RCTs with larger patient populations would potentially lead to more robust results and help to eventually reach more definitive conclusions on the topic.
